# Chemical Inactivation of *Bacillus subtilis* Endospores Preserves Recombinant Protein Antigenic Properties

**DOI:** 10.3390/microorganisms13112629

**Published:** 2025-11-19

**Authors:** Amalia A. Saperi, Atiqah Hazan, Nurfatihah Zulkifli, Hai Yen Lee, Sazaly AbuBakar

**Affiliations:** 1 Tropical Infectious Diseases Research and Education Centre (TIDREC), University Malaya, Kuala Lumpur 50603, Malaysia; aasaperi@um.edu.my (A.A.S.); atiqahazan@um.edu.my (A.H.); fatihahzulkifli@um.edu.my (N.Z.); leehaiyen@um.edu.my (H.Y.L.); 2 Institute of Advanced Studies, University Malaya, Kuala Lumpur 50603, Malaysia

**Keywords:** infectious diseases, *Bacillus subtilis*, spores, sporicidal, vaccine, chemical inactivation, biotechnology

## Abstract

Recombinant *Bacillus subtilis* endospores are promising bacterial expression platforms for oral protein delivery, such as oral vaccines. A simple and effective spore inactivation method that preserves protein functionality, however, is needed to prevent potential shedding into the environment. This study evaluated iron or copper combined with EDTA and ethanol as sporicidal solutions for the inactivation of recombinant spores expressing the *1PR82* gene. Immunoblot and immunofluorescence (IF) assay confirmed the presence of antigenic proteins post-treatment, while electron microscopy (SEM/TEM) assessed spore morphology. Mice immunization tested immunogenicity, and fecal analysis monitored gastrointestinal persistence. Iron ethanol treatment completely inactivated the spores while maintaining recombinant protein detection using antibody-based assays. SEM/TEM revealed morphological damage, yet antigenicity was preserved, as evidenced by robust IgG responses in immunized mice. Fecal analysis showed no prolonged spore shedding, confirming effective inactivation. These findings demonstrate that iron ethanol efficiently inactivates recombinant *B. subtilis* spores without compromising protein antigenicity. Despite structural damage, the recombinant protein remained immunogenic, and inactivated spores posed no environmental persistence risk. This inactivation method supports the safe use of *Bacillus subtilis* recombinant spores for oral delivery applications, balancing inactivation efficacy with functional protein preservation. Further research could optimize this approach for clinical or industrial applications.

## 1. Introduction

*Bacillus subtilis*, a bacterium generally regarded as safe (GRAS), is known for its ability to form resilient endospores under unfavorable growth conditions. These spores exhibit remarkable resistance to harsh environments, including the acidic pH of the stomach, enabling them to germinate and regenerate when conditions become favorable [1]. Due to this durability, GRAS spores are being investigated as potential oral delivery vehicles for recombinant proteins targeting the gut [2,3]. Additionally, *Bacillus subtilis* has gained attention as a probiotic, with studies demonstrating its ability to enhance gut health, modulate immune responses, exert antimicrobial effects, and stimulate digestive enzyme production [4]. However, the administration of live bacterial spores, especially those genetically engineered to express recombinant proteins, raises concerns about prolonged environmental persistence due to shedding. Thus, effective inactivation of *Bacillus subtilis* recombinant spores is highly desirable.

The extreme resilience of endospores against chemical agents that readily inactivate vegetative bacterial cells poses a significant challenge [5]. The efficacy of inactivation methods varies across sporulating bacterial species and depends on specific spore characteristics and treatment conditions. Common approaches for inactivating *Bacillus subtilis* spores include physical methods such as heat, radiation, ultraviolet (UV) light, ozone, and high-pressure processing (HPP), as well as chemical treatments like formaldehyde [6].

Heat treatment, typically involving autoclaving at 121 °C or higher, effectively inactivates spores by denaturing proteins and nucleic acids [6]. UV light can also achieve spore inactivation by inducing DNA damage, though its application is primarily limited to surface disinfection [7]. Ionizing radiation, while effective, is less practical due to its requirement for deep penetration [8]. Ozone gas inactivates spores through oxidative damage to cellular components and is commonly employed in water and air disinfection [9,10]. High-pressure processing (HPP), which applies pressures ranging from 100 to 1000 MPa, disrupts cellular structures and effectively inactivates spores while maintaining food quality [11,12]. Chemical agents such as ethylene oxide, peracetic acid, and formaldehyde can also inactivate *Bacillus subtilis* spores by damaging cell membranes, proteins, or nucleic acids [13,14,15].

While these methods, used individually or in combination, can achieve complete spore inactivation depending on sterilization or disinfection requirements, the inactivation of recombinant *B. subtilis* spores presents an additional challenge: preserving the functionality of the recombinant protein.

This critical limitation underscores the necessity for inactivation strategies that target spore viability while preserving protein functionality. A promising approach previously described by Kida et al. [16] demonstrated that dissolved metals in combination with EDTA and ethanol could efficiently inactivate wild-type *B. subtilis* spores. Here, we build upon this foundation to address a key translational gap. The study had three primary objectives: first, to assess the inactivation efficacy of the sporicidal solutions against recombinant spores expressing the *Acinetobacter baumannii* TonB-dependent receptor, second, to determine whether antigenicity was preserved following treatment, and third to establish the absence of environmental persistence under the tested conditions. Our results demonstrate not only efficient inactivation but, crucially, the retention of recombinant protein immunogenicity, thereby validating a safe and effective strategy for using inactivated recombinant spores as oral vaccine candidates.

## 2. Materials and Methods

### 2.1. Preparation of Recombinant B. subtilis Spores

Recombinant PHPS9 plasmid carrying the *Acinetobacter baumannii* TonB-dependent Receptor (TBDR) target gene *1PR82* was transformed into *Bacillus subtilis* as previously described [1,17]. Bacterial sporulation was induced by culturing the transformed bacteria in Glucose Yeast Extract (GYS) medium, and spores were harvested after 32 h [1,18]. The spore suspension was heat-treated in a water bath at 90 °C for 30 min to eliminate viable vegetative cells. Spores were then purified through sequential polyethylene glycol (PEG) precipitation and two-phase potassium buffer (SP Buffer) separation [19]. Following purification, spores were either chemically inactivated or maintained as untreated controls. The spore preparation was freeze-dried with the excipient (sodium chloride (Sigma Aldrich, St. Louis, MO, USA), Hy-case SF (Sigma Aldrich, St. Louis, MO, USA), ascorbic acid (Sigma Aldrich, St. Louis, MO, USA), inulin (Hi-media, Mubai, India), and magnesium stearate (Sigma Aldrich, St. Louis, MO, USA)) for storage and then reconstituted in an appropriate diluent (sodium bicarbonate (Sigma Aldrich, St. Louis, MO, USA), sodium carbonate (Sigma Aldrich, St. Louis, MO, USA), and inulin (Hi-media, Mubai, India) prior to use for in vivo studies. Spore pellets were stored at 4 °C, and freeze-dried spore preparations were stored at room temperature (20 °C).

### 2.2. Preparation of the Sporicidal Solutions

The sporicidal solutions consisted of 50 mM EDTA-2Na (ethylenediaminetetraacetic acid disodium salt) and 50 mM FeCl_3_.6H_2_O or 50 mM CuSO_4_.5H_2_O dissolved in a 0.85% NaCl solution (pH 1.1–1.4, adjusted with 10 M HCl). Subsequently, 50% ethanol was added to the mixture, and the pH was adjusted to 0.3 with 10 M HCl. The solutions were designated as the iron ethanol solution and the copper ethanol solution.

### 2.3. Recombinant Bacillus subtilis Spore Inactivation

Recombinant *Bacillus subtilis* spores were inactivated by suspending a 1:1 mixture of spore pellets and sporicidal solution in a glass bottle. The bottle was immersed in a shaking water bath maintained at 37 °C and shaken at 120 RPM for the required treatment duration. The efficacy of the sporicidal solutions was evaluated over four days and compared against a 4% formaldehyde solution and untreated controls. Spores were incubated with the iron ethanol solution for 2, 8, 12, or 24 h. The tests were repeated 2–3 times. Malachite green was used to stain the spores, and safranin red was used to stain vegetative cells for observation under light microscopy. The untreated recombinant spore samples used for light microscopy were analyzed using the diluted spore pellet (non-freeze-dried), while the inactivated recombinant spore samples were analyzed using the diluted freeze-dried spores.

### 2.4. Electron Microscopy of Inactivated Recombinant Bacillus subtilis Spores

Spore morphological changes were assessed using scanning electron microscopy (SEM) and transmission electron microscopy (TEM). Freeze-dried inactivated spores were applied to carbon tape for SEM observation. Untreated spores prepared in pellet form were placed on a 0.4 μm nuclepore membrane, fixed with 4% glutaraldehyde, and processed. The samples were coated with gold and were observed using an SEM instrument (FEI Quanta 650, Thermo Fisher Scientific, Waltham, MA, USA). TEM observation was performed on both untreated and inactivated samples in pellet form. The specimens were fixed with 4% glutaraldehyde, dehydrated through ethanol and acetone washes, embedded in epoxy resin, and sectioned into thin sections (50–100 nm). The samples were stained with heavy metal stains (uranyl acetate replacement and lead citrate), mounted onto TEM grids, and observed using a TEM instrument (Zeiss Libra 120, Carl Zeiss SMT AG, Oberkochen, Germany).

### 2.5. Immunoblot and Immunofluorescence of Inactivated Recombinant Bacillus subtilis Spores

For immunoblot detection, inactivated spores were serially diluted (neat to 1:10) and dot-blotted onto a nitrocellulose membrane using vacuum-assisted absorption. Once dried, the nitrocellulose membrane was blocked with 5% skim milk in TBS for 1 h at room temperature, followed by three washes with TBS-T (TBS + 0.1% Tween-20). Primary antibody incubation (1:1000 6×-His Tag monoclonal antibody, Invitrogen, Waltham, MA, USA) was performed overnight at 4 °C. After washing, membranes were incubated with HRP-conjugated goat anti-mouse IgG secondary antibody (1:5000, Abcam, Cambridge, UK) for 1 h at 37 °C. Protein detection was achieved using Clarity Western ECL substrate (Bio-Rad, Hercules, CA, USA) and imaged with a Gel Doc system (Bio-Rad). For immunofluorescence, spores were probed with anti-His-Tag antibody followed by Alexa Fluor 488-conjugated goat anti-mouse IgG (Abcam, Cambridge, UK). Fluorescent signals were visualized using appropriate filter sets.

### 2.6. Immunogenicity of Inactivated B. subtilis Spores Expressing A. baumannii TBDR

Female BALB/c mice (8–12 weeks old, *n* = 7/group) were orally administered either diluent (control), *B. subtilis* spore control, or inactivated *B. subtilis* spores expressing *A. baumannii* TBDR (1 × 10^11^ CFU/mL) in three immunizations. Each immunization consisted of three daily inoculations (immunization 1: days 1–3; immunization 2: days 17–19; immunization 3: days 33–35). Blood was collected by retro-orbital puncture 14 days after each immunization (days 17, 33, and 49), and serum was isolated by centrifugation (1200× *g*, 4 °C, 10 min).

For ELISA, Flat-bottom 96-well polystyrene plates (Nunc MaxiSorp, Thermo Fisher Scientific, Waltham, MA, USA) were coated with inactivated recombinant spores (5 × 10^5^ CFU/well), blocked with 1% BSA (SeraCare, Milford, MA, USA), and diluted serum (1:100) was added in duplicates and incubated overnight at 4 °C. ELISA was performed strictly as described by the kit manufacturer (Bio-techne, Minneapolis, MN, USA), and results were baseline-corrected against the control (diluent) group. Normalization was performed using GraphPad Prism Version 9.0.0 (GraphPad Software Inc., San Diego, CA, USA) after calculating the mean and SEM from *n* = 7/group (serum).

### 2.7. Recovery of Inactivated Recombinant Bacillus subtilis Spores in BALB/c Mice Feces

Mice were divided into four groups: Group 1 (diluent control), Group 2 (*B. subtilis* spore control), Group 3 (untreated *B. subtilis* expressing *A. baumannii* TBDR), and Group 4 (inactivated *B. subtilis* expressing *A. baumannii* TBDR). Bacterial spores in 200 μL solution were administered to the mice over three consecutive days. Fecal samples were collected on days 1, 2, 3, and 4 post-inoculation, homogenized, and plated on selective agar (2× YT, HiMedia, Mumbai, India) supplemented with 5 µg/µL chloramphenicol.

### 2.8. Statistical Analysis

Data were plotted and analyzed using GraphPad Prism Version 9.0.0 (GraphPad Software Inc., San Diego, CA, USA) and are presented as means and standard error of mean (SEM). The significance of the data was determined using the ordinary one-way ANOVA. Differences were considered significant when *p* < 0.05.

## 3. Results

### 3.1. Inactivation of the B. subtilis Spores Expressing A. baumannii TBDR

**Figure 1:**
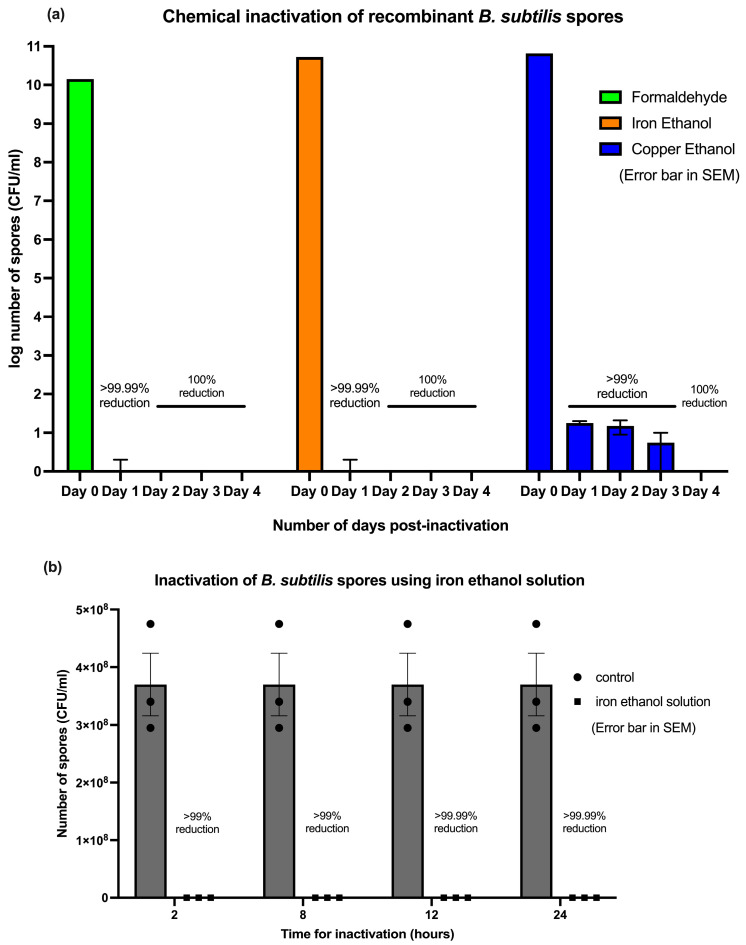
Inactivation of the recombinant *Bacillus subtilis* spores after incubation with formaldehyde, iron ethanol, or copper ethanol. (**a**) Recombinant *Bacillus subtilis* spores incubated with formaldehyde (spore inactivation control), iron ethanol solution, or copper ethanol solution were observed from day 0 to day 4 for inactivation. (**b**) Spores incubated with iron ethanol were sampled at 2, 8, 12, and 24 h to determine the amount of time needed to inactivate the spores using the iron ethanol solution. Error bars are presented in SEM.

Earlier work of Kida et al. demonstrated the efficacy of the iron–copper ethanol sporicidal solution against wild-type *Bacillus subtilis* spores. Building on this study, here we validated its application for recombinant spores by employing the established combination treatments and benchmarking their performance against the conventional formaldehyde inactivation. The initial bacterial spore counts prior to inactivation were determined to be at 1.41 × 10^10^ CFU/mL for the 4% formaldehyde treatment group, 5.30 × 10^10^ CFU/mL for the iron ethanol solution treatment group, and 6.6 × 10^10^ CFU/mL for the copper ethanol solution treatment group. Following the inactivation, treatment with 4% formaldehyde and iron ethanol formulations resulted in complete spore inactivation for 1 (1 cells/mL, >99.999% reduction, *p* < 0.0001), 2 (0 cells/mL, 100% reduction, *p* < 0.0001), 3 (0 cells/mL, 100% reduction, *p* < 0.0001), and 4 (0 cells/mL, 100% reduction, *p* < 0.0001) days treatment exposure. Spores incubated with copper ethanol showed <100% inactivation for the 1 (15 cells/mL, >99% reduction, *p* < 0.0001), 2 (11.5 cells/mL, >99% reduction, *p* < 0.0001), and 3 (4.5 cells/mL, >99% reduction, *p* < 0.0001) day treatment exposure, and achieved complete inactivation (100%) after a 4 (0 cells/mL, 100% reduction, *p* < 0.0001) day treatment (Figure 1a).

Using the iron ethanol solution, the time required for the inactivation of recombinant *B. Subtilis* spores was identified (Figure 1b). After 2 h of treatment, the mean spore count decreased from 3.7 × 10^8^ CFU/mL in the control group to 2.72 × 10^1^ CFU/mL in the treatment group, suggesting a <100% reduction (*p* = 0.0024). At 8 h, the mean spore count decreased from 3.7 × 10^8^ CFU/mL in the control group to 1.95 × 10^1^ CFU/mL in the treatment group (*p* = 0.0024). Similarly, at 12 h, the mean spore count decreased from 3.7 × 10^8^ CFU/mL in the control group to 8.7 × 10^0^ CFU/mL in the treatment group (*p* = 0.0024), and at 24 h, the mean spore count decreased from 3.7 × 10^8^ CFU/mL in the control group to 5.83 × 10^0^ CFU/mL in the treatment group (*p* = 0.0024).

### 3.2. Morphological Characterization of Inactivated B. subtilis Spores Expressing A. baumannii TBDR

**Figure 2:**
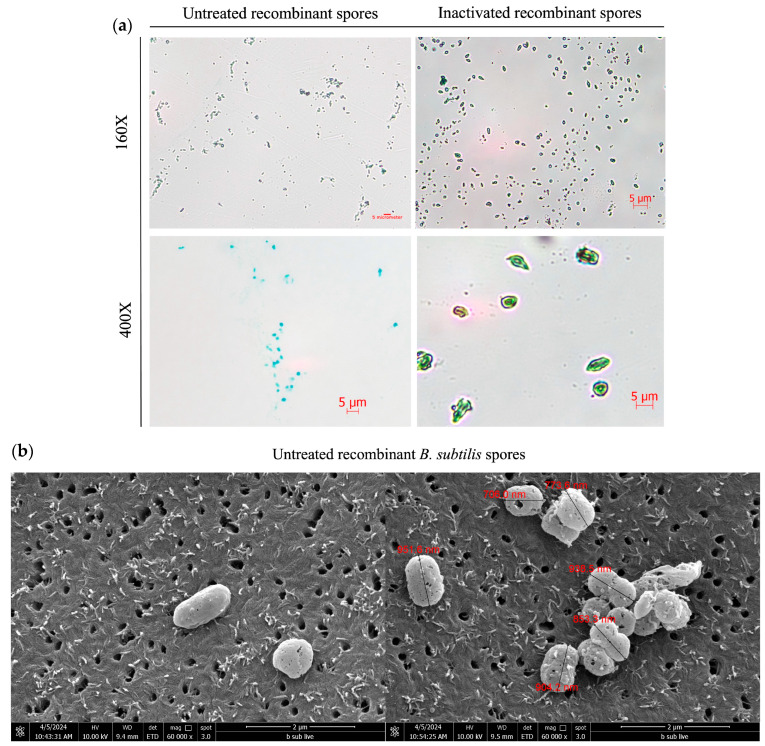

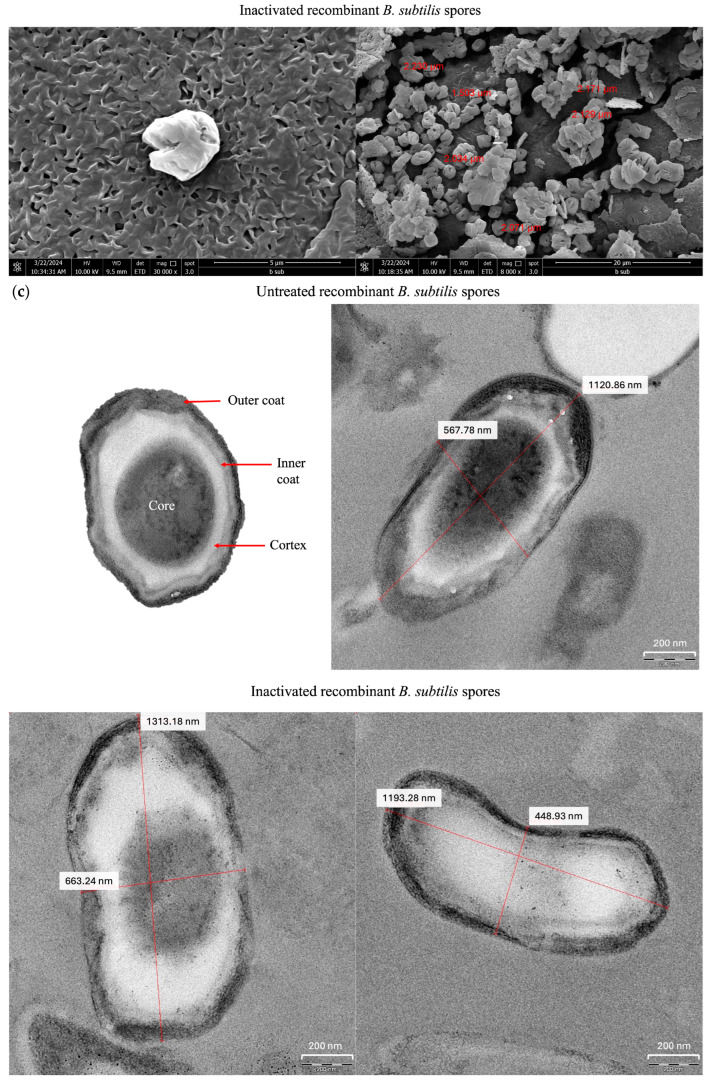
Microscopic observation of inactivated recombinant *Bacillus subtilis* spores TBDR. (**a**) Untreated and inactivated recombinant *Bacillus subtilis* spores were observed under the light microscope at 400× magnification following the staining with malachite green (spore) and safranin red (vegetative cell). (**b**) Untreated *B. subtilis* spores were placed on a 0.2 μm nuclepore membrane and were observed under the SEM at 60,000× magnification and inactivated spores in freeze-dried form were placed on the copper tape and observed under the SEM at 30,000× and 8000×. (**c**) Untreated and inactivated *B. subtilis* spores were observed under the TEM at 20,000× magnification.

*B. subtilis* spores were observed under light microscopy, scanning electron microscopy (SEM), and transmission electron microscopy (TEM). Under a light microscope, untreated spores were oval-shaped and measured approximately 1–2 µm in size, appearing green in color when stained with malachite green, while inactivated spores appeared marginally larger and displayed a disheveled morphology (Figure 2a). Under SEM, untreated spores were spherical or ellipsoidal with a size of 1–2.5 µm, while the inactivated spores appeared similar in shape to the untreated *B. subtilis* spores but presented a shriveled or deformed morphology (Figure 2b). TEM revealed that the untreated spores presented with an outer coat, inner coat, cortex, and core, typical of *B. subtilis* spores, while, the inactivated spores were 0.7–1.5 µm in size and presented a thinner outer coat, lacked an inner coat and cortex, and had a deformed core, with many appearing as ‘ghost-like’ spores without the core (Figure 2c).

### 3.3. Surface Protein Detection of Inactivated B. subtilis Spores Expressing A. baumannii TBDR

**Figure 3:**
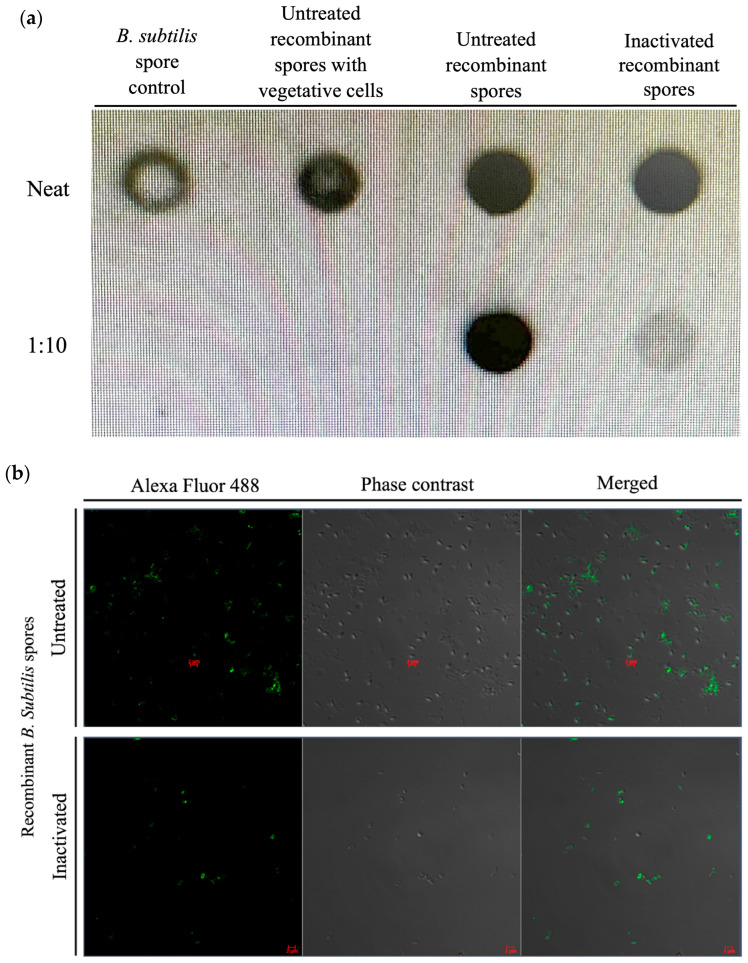
Detection of inactivated recombinant *B. subtilis* spores by immunoblot (dot blot) and immunofluorescence staining. (**a**) The surface display of the recombinant protein on untreated and inactivated *B. subtilis* spores expressing *A. baumannii* TBDR was evaluated using an immunoblot (dot blot) assay and immunofluorescence microscopy. *B. subtilis* spore control (carrying non-recombinant plasmid PHPS9), untreated recombinant spores with vegetative cells before removing residual vegetative cells, untreated recombinant spores, and inactivated recombinant spores, were diluted from 0 to 1:10 and observed by immunoblotting to demonstrate the antibody binding to the recombinant protein (6×-His tag). (**b**) Untreated spores and inactivated spores were observed by immunofluorescence detection with Alexa Fluor 488 (green dye).

The immunoblot (dot blot) results showed the detection of the protein of interest (Figure 3a) using the specific antibody against the recombinant protein detected with anti-6× His in both the untreated and inactivated recombinant spore preparation compared with the control spores (non-recombinant spores) at undiluted and 1:10 dilutions for both the untreated and inactivated recombinant spores. Immunofluorescence staining of the untreated and inactivated recombinant spores demonstrated the detection of the recombinant protein in the form of green fluorescence on the surface of the spores (Figure 3b).

### 3.4. Immunogenicity of Inactivated B. subtilis Spores Expressing A. baumannii TBDR

**Figure 4:**
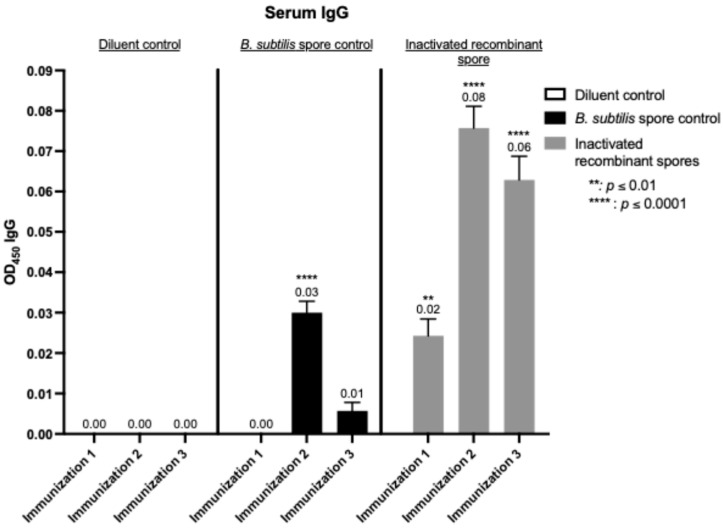
Detection of spore-specific IgG in serum of mice after inoculation with *B. subtilis* spores expressing *A. baumannii* TBDR.

The immunogenicity of inactivated *B. subtilis* spores expressing *A. baumannii* TBDR was assessed by determining the recombinant protein-specific IgG levels in serum using ELISA (Figure 4). After the first immunization, IgG levels showed a slight but significant increase (0.02 nm; *p* < 0.0049). A more pronounced rise was observed following the second immunization (0.08 nm, *p* < 0.0001), a trend that persisted after the third immunization (0.06 nm, *p* < 0.0001).

Serum from BALB/c mice (*n* = 7/group) was analyzed using ELISA to measure TBDR spore-specific IgG antibody measured by wavelength (OD450) following inoculation with diluent control, *B. subtilis* spore control, and inactivated *B. subtilis* spores expressing *A. baumannii* TBDR protein. Measurements were taken 14 days after (a) immunization 1, 2, and 3.

### 3.5. Recovery of Inactivated Recombinant Bacillus subtilis Spores in BALB/c Mice Feces

Fecal samples collected from mice administered with either untreated or inactivated recombinant *Bacillus subtilis* spores were plated on selective agar consisting of chloramphenicol, and the recombinant plasmid consisted of the chloramphenicol resistance gene (Table 1). Feces collected on day 1 post-inoculation from mice that were orally fed with untreated recombinant spores showed too numerous to count (TNTC) number of bacterial colonies. No bacterial colonies were detected in the feces of the group that received inactivated recombinant *Bacillus subtilis*. On day 2, the fecal samples from the untreated recombinant spore group showed TNTC bacterial colonies, while no bacterial colonies were found in the inactivated recombinant *B. subtilis* spore group. By day 3, the bacterial colony count in the feces of mice given the untreated spores was 2.2 × 10^2^ CFU/mL, with none detected in the inactivated recombinant *B. subtilis* spore group. By day 4, no detectable bacterial colonies were present in any of the groups.

## 4. Discussion

The development of effective and safe methods for inactivating recombinant *Bacillus subtilis* spores while preserving their functional proteins is crucial for their application in oral delivery applications of the bacterial spores. In this study, we demonstrate that two sporicidal solutions, comprising 50 mM EDTA-2Na with either 50 mM FeCl_3_·6H_2_O or 50 mM CuSO_4_·5H_2_O in a 0.85% NaCl and 50% ethanol solution, efficiently inactivated *B. subtilis* spores. The iron ethanol formulation achieved complete inactivation by day 2, whereas the copper ethanol solution required four days. Electron microscopy revealed morphological alterations, including spore coat thinning and core disruption, suggesting these structural changes potentially contributed to the inactivation mechanism. There was also the observed “ballooning” effect seen in the freeze-dried spore samples, which could be a result of the formation of large ice-crystals due to rapid freezing during the freeze-drying process [20]. This is likely because the inactivated spores from pellets retained their original size, whereby spore size for both groups falls well within the expected size range for *Bacillus subtilis* spores (1.0–1.5 µm) under TEM (Figure 2c). Despite these morphological changes, results from the immunoblot and immunofluorescence assays are in congruence with the suggestion that the recombinant protein retained its antigenicity. Furthermore, the inactivated spores elicited specific and escalating immune responses in mice, as evidenced by elevated serum IgG levels specific to the *A. baumannii* TBDR antigen following each immunization, further supporting the preservation of the recombinant protein immunogenicity.

The inactivation mechanisms of the sporicidal solutions likely involve synergistic interactions among the solution components. EDTA, a chelating agent, may weaken the spore coat by extracting essential divalent cations, enhancing permeability to metal ions [16]. Subsequent exposure to Fe^3+^ or Cu^2+^ could exacerbate structural damage through membrane disruption [21] and DNA binding [22,23]. Ethanol, while ineffective against intact spores alone, may further destabilize membranes after metal ion penetration [24]. Additionally, the acidic environment of the sporicidal solution may impair spore-associated enzymes and metabolic processes [6], hence, collectively leading to irreversible damage, as indicated by the observed “ghost-like” spores lacking intact cores. Unlike traditional inactivation methods (e.g., formaldehyde or heat), which denature the spore proteins [25,26,27], treatment with the sporicidal solutions as described here preserves antigenic epitopes of the recombinant protein. While recombinant spores have been used as protein delivery platforms [2], and inactivated *B. subtilis* spores were used as heterologous boosters [28], the present study represents the first to report an in vitro validation of the iron ethanol sporicidal solution for recombinant spores and demonstrated retained detection of the recombinant protein expressed on the inactivated *B. subtilis* spores. In addition, results from the present study demonstrated no persistent shedding of the inactivated spores upon oral delivery to mice within 24 h post-immunization—the inactivated spores could not germinate. This observation, hence, addresses the concern that recombinant *Bacillus subtilis* spores could continuously be released into the environment.

The present study was undertaken with a defined translational objective: to adapt an established sporicidal method for wild-type *B. subtilis* spores and validate its applicability to recombinant spores expressing a heterologous antigen. The experimental scope, therefore, centered on benchmarking iron or copper ethanol sporicidal solutions against a standard agent (formaldehyde), with particular emphasis on demonstrating the retention of surface antigen detectability and immunogenicity following inactivation. In line with this focused design, controls for individual solution components (e.g., ethanol, iron, or copper alone) were not included, as the sporicidal activity of the complete formulation has already been conclusively demonstrated by Kida et al. [16]. Our intent was not to dissect the underlying mechanisms but to extend the optimized mixture to a novel biological context. While this approach successfully achieved its primary objective, it necessarily constrains mechanistic interpretations: the relative contribution of each component to the overall sporicidal effect remains unresolved, representing a limitation that future studies should address.

In addition, further characterization is needed to elucidate the molecular interactions between metal ions and spore components, for example, through metal uptake kinetics or DNA damage assays, which would offer deeper mechanistic insight beyond the observed structural findings from TEM. Finally, although separate immunological and murine fecal shedding data support the preservation of antigenicity and absence of spore shedding, the full translational development of this platform will require further toxicological evaluation in higher animal models to exclude any potential residual effects of metal components, such as unintended inflammatory or immunological responses. We believe that the defined parameters of this study were appropriate to establish essential in vitro proof-of-concept, creating a robust foundation for subsequent in vivo studies and further mechanistic investigation.

In summary, the present study establishes a novel, food-grade strategy for inactivating recombinant *B. subtilis* spores while preserving protein functionality. The iron and copper ethanol solutions described here offer distinct advantages over traditional inactivation methods—avoid the use of harsher chemicals and maintain recombinant protein antigenicity. This approach has broader implications, including enabling oral delivery of heat-sensitive antigens in oral vaccine development, inactivation of paraprobiotics for use in immunocompromised individuals, and providing industrial applications in fermentation and food safety.

## 5. Conclusions

This study successfully validates the adaptation of an iron ethanol sporicidal solution for recombinant *B. subtilis* spores, establishing it as a viable strategy that balances efficient inactivation with the crucial antigen detection and immunogenicity. While the precise molecular mechanisms warrant further elucidation, our findings confirm that this approach mitigates the key environmental risk of recombinant spore shedding and maintains immunogenicity, thereby addressing critical barriers for the usage of recombinant *B. subtilis* spores for oral consumption. The evidence presented provides a robust in vitro and pre-clinical foundation, positioning this inactivation platform as a promising, food-grade alternative to conventional methods and paving the way for its future application in oral vaccine delivery and other biotechnological fields.

## 6. Patents

The reported work has been submitted to the Intellectual Property Corporation of Malaysia (MyIPO) for patent filing with patent filing number: PI2024005912.

## Figures and Tables

**Table 1:** Number of *Bacillus subtilis* colonies (CFU/mL) recovered on the selective agar from the fecal pellet of mice fed with recombinant *B. subtilis* spores.

Groups	Number of Recombinant *B. subtilis* Recovered (CFU/mL)			
	Day 1	Day 2	Day 3	Day 4
Diluent control	0	0	0	0
*B. subtilis* spores control	0	0	0	0
Untreated recombinant *B. subtilis* spores	TNTC	TNTC	2.2 × 10^2^	0
Inactivated recombinant *B. subtilis* spores	0	0	0	0

TNTC: Too numerous to count.

## Data Availability

The original contributions presented in this study are included in the article. Further inquiries can be directed to the corresponding author.

## Abbreviations

The following abbreviations are used in this manuscript:

*A. baumannii*	*Acinetobacter baumannii*
*B. subtilis*	*Bacillus subtilis*
BAL	Broncheoalveolar Lavage
BSA	Bovine Serum Albumin
CFU	Colony Forming Unit
EDTA	Ethylenediaminetetraacetic acid
ELISA	Enzyme-linked Immunosorbent Assay
GRAS	Generally Regarded as Safe
IgG	Immunoglobulin G
MDR	Multidrug Resistant
PEG	Polyethylene Glycol
TBDR	TonB Dependent Receptor
